# Screening disease feature genes and analyzing correlations with immune cell infiltration in knee osteoarthritis chondrocytes based on multiple machine learning algorithms

**DOI:** 10.1371/journal.pone.0351666

**Published:** 2026-06-16

**Authors:** Jing-le Zhuge, Xi-yong Li, Yong-le Wang, Juan-fen Ma

**Affiliations:** Department of Orthopaedics, Wenzhou TCM Hospital Of Zhejiang Chinese Medical University, Wenzhou, P.R. China; University of Sharjah, UNITED ARAB EMIRATES

## Abstract

**Objective:**

This study aimed to comprehensively analyze differentially expressed genes (DEGs) in chondrocytes from patients with knee osteoarthritis (OA) by integrating multiple machine learning algorithms and bioinformatics techniques, to unravel the underlying molecular mechanisms associated with OA chondrocytes, and to provide novel insights for the innovation of clinical therapeutic strategies.

**Methods:**

We downloaded the GSE117999, GSE114007, GSE169077, GSE246425, and GSE178557 datasets from the public Gene Expression Omnibus (GEO) database as the training set, while GSE57218 served as an independent validation set. To ensure data consistency and comparability, the training set was normalized, and the ComBat algorithm was applied to eliminate batch effects, yielding a merged gene expression dataset. Subsequent differential expression analysis was performed to identify genes with significant changes under disease conditions, followed by enrichment analysis. To more accurately identify genes closely linked to disease characteristics, we independently analyzed the merged dataset using three machine learning algorithms: Lasso regression, random forest, and support vector machine (SVM). The intersection of results from these three methods was used to construct a robust list of disease-related feature genes. These prominent feature genes were validated in the training set and further externally confirmed using the GSE57218 dataset. Additionally, the CIBERSORT algorithm was employed to quantify immune cell infiltration in the normalized gene expression data, selecting infiltration results with high reliability (P < 0.05). Focusing on the target genes, we clarified the strength and significance of their associations with immune cell infiltration levels, comprehensively revealing differences in immune cell infiltration profiles between groups and the potential associations with target genes.

**Results:**

*DDIT3* and *PFKFB3* were significantly downregulated in OA patients. *DDIT3* was specifically associated with lipid metabolism, apoptosis, and inflammatory genes (e.g., *TNFRSF12A*), whereas *PFKFB3* was linked to phospholipid synthesis and cell cycle genes (e.g., *CHKA*). Both genes were associated with core OA-related pathways, including PI3K-Akt and AGE-RAGE. Immune infiltration analysis revealed that *DDIT3* was positively correlated with pro-inflammatory mast cells and M1 macrophages, while *PFKFB3* was negatively correlated with activated dendritic cells. Collectively, these two genes were associated with immune cell infiltration patterns. The competing endogenous RNA (ceRNA) network analysis indicated that *DDIT3* was associated with axes such as *LINC00689-miR-769-5p*, and *PFKFB3* was associated with complex networks like *GAS6-AS1-miR-146a-5p.*

**Conclusion:**

*DDIT3 and PFKFB3 are key candidate genes associated with the pathological progression of OA. Their downregulation is correlated with inflammatory and metabolic disturbances in chondrocytes, supporting their potential use as diagnostic biomarkers and therapeutic targets for OA.*

## Introduction

OA is a globally prevalent chronic joint disease, characterized by core pathological features including articular cartilage degeneration, abnormal remodeling of subchondral bone, synovitis, and marginal osteophyte formation [1]. It severely impairs patients’ daily activities and quality of life. With the intensification of global population aging, the incidence of OA continues to rise, particularly among the elderly. It has become one of the major causes of chronic pain and motor dysfunction in the elderly, imposing a heavy medical burden and challenges on public health systems worldwide [2,3].

The pathogenesis of OA is complex and not yet fully elucidated, involving the interaction of multiple factors such as genetic susceptibility, aging, obesity, mechanical injury, abnormal activation of inflammatory factors, and metabolic disorders [4]. Previous studies have mostly focused on chondrocyte degeneration itself, but in recent years, accumulating evidence has indicated that immune cell infiltration plays a crucial role in the occurrence and progression of OA [5]. The imbalance of the local joint immune microenvironment—such as the abnormal accumulation and activation of immune cells including macrophages, T cells, and neutrophils—leads to the continuous release of inflammatory factors. These factors not only directly exacerbate synovitis but also accelerate the secretion of cartilage matrix-degrading enzymes, disrupt chondrocyte homeostasis, and thereby promote cartilage degeneration and abnormal remodeling of subchondral bone, forming a “inflammation-injury” vicious cycle. Moreover, the degree of immune infiltration is closely associated with the pain intensity and the rate of joint destruction progression in OA patients [6]. However, the specific associations between immune infiltration and the pathological processes of OA, as well as the associations between key genes and immune cell infiltration, still need further exploration.

The rapid development of bioinformatics technology has provided a new approach for investigating the immune-related mechanisms of OA. By leveraging high-throughput sequencing data and bioinformatics analysis tools, OA-related gene expression datasets can be obtained from public databases. After normalization to eliminate batch effects, DEGs under disease conditions can be identified through differential expression analysis [7]. Subsequently, algorithms such as CIBERSORT can be used to quantitatively analyze the characteristics of immune cell infiltration in joint tissues and screen out highly reliable infiltration results [8]. Further exploration of the strength and significance of associations between core genes and immune cell infiltration levels enables the systematic revelation of differential features of immune infiltration in OA and potential association networks [9].

This study intends to employ bioinformatics methods. Based on multiple OA-related training and validation sets, we will first complete data preprocessing and differential gene screening, then focus on analyzing the immune microenvironment characteristics of OA through immune cell infiltration analysis. Meanwhile, a combination of various machine learning algorithms will be used to screen and validate core genes associated with OAimmune status. It is expected that this study will clarify the associations between immune infiltration and OA, provide a scientific basis for the development of early diagnostic biomarkers and the formulation of immune-targeted therapeutic strategies for OA, and contribute to the advancement of precision medicine in OA.

## Materials and Methods

### Data Acquisition

This study primarily utilized microarray technology for gene expression analysis. Gene expression datasets of chondrocytes from patients with OA and normal individuals were retrieved from the public GEO database on October 15, 2025. The data used are publicly available and de-identified, with no individually identifiable information. Ethics statement: This study uses publicly available data from GEO, involves no human subjects, and therefore does not require ethical approval. We searched and extracted datasets from the GEO database using the following keywords: “OA”, “chondrocytes”, “microarray”, “human samples”, and corresponding disease-specific gene expression patterns. The final included datasets were GSE117999, GSE114007, GSE169077, GSE246425, GSE178557, and GSE57218. Sample sizes were: GSE117999 (12 OA + 12 control), GSE114007 (20 OA + 18 control), GSE169077 (6 OA + 5 control), GSE246425 (4 OA + 8 control), GSE178557 (8 OA + 4 control), and GSE57218 (33 OA + 7 control). Due to data collection limitations, detailed age and gender information were unavailable for all samples. Although age and gender are potential confounding factors, this study focused on the expression patterns of DEGs in OA chondrocytes, their associations with disease progression, and associations with immune cell infiltration. The available data sufficed to meet research objectives, so these datasets were included for subsequent analyses.

### Data Preprocessing and Batch Effect Correction

To ensure analytical accuracy and reliability, we first performed rigorous data preprocessing: genes (rows) or samples (columns) with excessive missing values were excluded to purify the dataset; duplicate genes were merged by taking the mean expression value to streamline the workflow and reduce information redundancy; data were standardized using the normalizeBetweenArraysfunction from the limmapackage [7] to eliminate inter-experimental systematic biases and ensure cross-sample comparability; batch effects introduced by different experimental platforms during multi-dataset integration were corrected using the R package “sva” [10] (specifically applied to the five training sets: GSE114007, GSE117999, GSE169077, GSE178557, and GSE246425); and after correction, datasets were merged by consistent disease status to establish a high-quality foundation for subsequent analyses.

### Identification of DEGs

To systematically identify DEGs between OA patients’ chondrocytes and normal controls, we performed differential expression analysis on the merged expression matrix of the 5 batch-corrected GEO datasets: first, we used the limma package to screen DEGs from batch-corrected data, then further validated results with DESeq2 [11]—a classic R tool for count data that fits expression to a negative binomial distribution and employs the Wald test for inter-group differences, ensuring reliability. We set a strict screening criterion: adjusted P < 0.05 and log₂ fold change (log₂FC) ≥ 1, balancing statistical and biological significance. Identified DEGs were sorted by log₂FC (descending), and the top 50 up/downregulated genes (or all if <50) were selected. Heatmaps (generated via pheatmap) visualized row-normalized expression patterns, with each sample’s GEO source and group labeled at the top to intuitively present DEG characteristics between groups and lay groundwork for mining OA-related core genes.

### Enrichment Analysis

To explore the biological functions of significantly DEGs, we used the R package clusterProfilerto perform Gene Ontology (GO) and Kyoto Encyclopedia of Genes and Genomes (KEGG) pathway enrichment analyses [12]. We set a P-value < 0.05 as the threshold to identify statistically significant biological processes (BP), cellular components (CC), molecular functions (MF), and key signaling pathways.

### Screening of Disease-Related Feature Genes

Machine Learning-Based Feature Selection To identify robust OA-associated core genes, we integrated three complementary machine learning algorithms: LASSO regression, Random Forest (RF), and Support Vector Machine-Recursive Feature Elimination (SVM-RFE). All analyses were performed in R (v4.3.2). First, LASSO regression was applied using the glmnet package to compress redundant features via L1 regularization. The optimal regularization parameter (λ) was determined via 10-fold cross-validation. To ensure reproducibility and mitigate partition bias, a fixed random seed (set.seed(123)) was set prior to data splitting, and the λ yielding the minimum mean cross-validated error across the 10 folds was selected. Genes retaining non-zero coefficients at this λ were retained. Second, a RFmodel comprising 500 decision trees was constructed using the randomForest package. Feature importance was ranked based on the Mean Decrease Gini index, and genes with an importance score >2 were selected as high-discriminative candidates. Third, SVM-RFE was implemented via the e1071 package to recursively eliminate low-contribution features. Feature elimination was guided by a 10-fold cross-validation loop that minimized classification error at each iteration. The final core gene set was defined as the intersection of genes identified by all three methods, ensuring high reliability through algorithmic consensus. The cross-validation procedures were implemented as standard 10-fold partitions; procedural stability was rigorously controlled through fixed random seeds, and the robustness of the selected features was further validated in an independent external cohort (GSE117999).

### Validation of Disease-Related Feature Genes in the Training Set

We used the batch-corrected and merged gene expression dataset of the training set (which included disease-related differentially expressed feature genes). We processed the expression data and gene lists via the R package limma, and screened effective target genes through intersection matching. Violin plots were generated with ggpubr, and between-group statistical tests were performed using the stat_compare_meansfunction (significance threshold: P < 0.05; notation: ***P < 0.001, ** P < 0.01, * P < 0.05, ns = not significant) to validate differential gene expression. receiver operating characteristic (ROC) curves were plotted with pROC to calculate AUC values (with 95% confidence intervals (CI)), optimal cutoffs, and metrics like sensitivity and specificity—assessing the genes’ diagnostic performance for the disease.

### Validation of Disease-Related Feature Genes in the Validation Set

To validate the reliability of results from the training set, we included the disease-related feature genes (identified as the intersection of three algorithms) in the normalized gene expression dataset of the validation set. We processed the data and screened for effective genes using the R package limma. Violin plots were generated with ggpubrto visualize gene expression differences, and between-group Wilcoxon tests were performed via stat_compare_means(significance threshold: P < 0.05; notation: *** P < 0.001, ** P < 0.01, * P < 0.05, ns = not significant) to confirm consistency in differential expression. ROC curves were constructed using pROCto calculate Area Under the Curve (AUC) values (with 95% CI), optimal cutoffs (derived from the Youden index), and metrics like sensitivity and specificity. We selected genes with high diagnostic efficacy using a criterion of AUC > 0.7.

### Differential Expression Analysis and Co-expression Network Construction Based on Core Gene Stratification

To explore differences in gene expression profiles between high- and low-expression states of core genes, we selected samples from the merged normalized expression dataset as research subjects. We divided samples into high- and low-expression groups based on the median expression of each core gene. Using the limmapackage, we performed differential expression analysis between the two groups, setting screening thresholds as |logFC| > 1 and adjusted P-value < 0.05. We identified DEGs and saved the results. Heatmaps generated via the pheatmappackage displayed the top 20 up- and downregulated genes, intuitively showing clustering characteristics of gene expression in the two groups. Furthermore, we extracted expression matrices for core genes and DEGs, constructed correlation matrices, and plotted correlation heatmaps using the corrplotpackage. Positive correlations (red gradient) and negative correlations (blue gradient) between genes were visualized with circles, showing co-expression patterns between core genes and other DEGs.

### Immune Cell Infiltration and Correlation Analysis

We used the CIBERSORT algorithm combined with the LM22 reference signature matrix to quantify immune cell infiltration proportions in the merged and normalized gene expression dataset. Although the utilized GEO datasets were initially annotated as “chondrocyte datasets,” verification of the original sample collection and RNA extraction protocols confirms that the material represents full-thickness knee cartilage tissue explants or surgical biopsies, rather than flow-sorted purified chondrocytes. In osteoarthritis, cartilage constitutes a heterogeneous microenvironment containing not only chondrocytes but also stromal cells, vascular components, and both resident and infiltrating immune populations (e.g., macrophages, T cells, mast cells), particularly during disease progression. Therefore, deconvolution analysis of these bulk cartilage transcriptomes is biologically appropriate and aligns with established OA immunophenotyping literature. To ensure analytical rigor, we conducted 1,000 permutation tests to validate result reliability and retained only samples with a CIBERSORT deconvolution P < 0.05 for downstream analyses. The relative infiltration proportions of 22 immune cell subtypes were subsequently visualized and statistically compared using R packages (reshape2, ggpubr, and corrplot). Grouped bar charts illustrated infiltration distribution differences between control and OA groups, while box plots combined with Wilcoxon rank-sum tests (significance threshold: P < 0.05; **P < 0.001, *P < 0.01, P < 0.05) systematically compared infiltration levels across groups.

### Correlation Analysis Between Core Genes and Immune Cell Infiltration

To determine the associations between the identified core genes and immune cell infiltration, we integrated gene expression data with the filtered immune cell proportion matrices from the experimental group samples. Spearman’s rank correlation analysis was performed to evaluate these relationships, with statistical significance defined as P < 0.05. For significantly correlated immune cell subsets, we generated scatter plots (incorporating trend lines and marginal density distributions) and correlation lollipop plots to visualize the strength and direction of associations, thereby facilitating the identification of key immune cell–gene interactions in the OA cartilage microenvironment.

### Construction of the ceRNA Network for Core Genes

We predicted target genes of core genes using three databases: miRanda, miRDB, and TargetScan. We selected high-confidence miRNAs commonly predicted by all three databases. Next, using the spongeScan database, we identified lncRNAs that sponge these miRNAs via the ceRNA mechanism. Finally, we integrated interactions among core genes, high-confidence miRNAs, and associated lncRNAs to construct the ceRNA network.

### Statistical Methods, Software, and Tools

We used R as the core analysis platform. The limmaand clusterProfilerpackages were employed for gene expression processing and differential analysis [13]. We visualized results using packages such as ggpubrand ggplot2. The pROCpackage evaluated gene diagnostic efficacy, and the CIBERSORT algorithm quantified immune cell infiltration. Statistical methods included Wilcoxon rank-sum test, limma linear model, Spearman’s rank correlation test, ROC curve analysis, and principal component analysis (PCA). A P-value < 0.05 was considered statistically significant. For differential gene screening, thresholds were set as |logFC| > 1 and adjusted P-value < 0.05 (False Discovery Rate (FDR) correction). Key analytical parameters:CIBERSORT: LM22 matrix, 1000 permutations;LASSO/RF/SVM-RFE: 10-fold cross-validation with fixed random seed;Multiple testing: Benjamini–Hochberg FDR correction.

## Results

### Data Processing

We first preprocessed, organized, and merged the five datasets to yield a combined gene expression matrix for the training set (containing 11,980 expression data points). Meanwhile, we processed the GSE57218 dataset to generate an independent validation set with a gene expression matrix of 37,791 data points. To evaluate batch effect correction, we performed PCA on gene expression distributions across the five datasets (including GSE114007) before and after correction. Prior to correction, samples clustered distinctly by dataset (GSE accession), indicating significant batch effects; after correction, batch-specific clustering was markedly attenuated, with samples dispersing more evenly and aligning better with biological traits. These results confirmed effective elimination of batch effects, providing a reliable foundation for subsequent analysis of the merged dataset.

### Differential Expression Analysis

**Fig 1:**
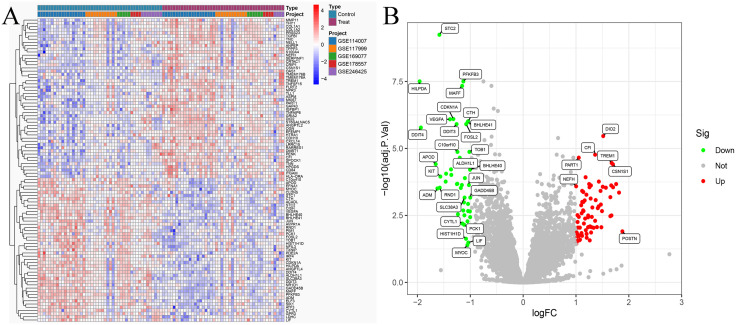
Heatmap and Volcano Plot of DEGs in the OA Training Set. (A) Heatmap of DEGs. **(B)** Volcano plot of DEGs. Red indicates upregulated genes; blue indicates downregulated genes. “Control” denotes healthy chondrocytes; “Treat” denotes OA chondrocytes. Differential expression was analyzed using the limma R package, with thresholds set at adjusted P < 0.05 and |log₂ fold change (FC)| > 1. Note: Column ordering is driven by hierarchical clustering of gene expression profiles, which may alter the visual grouping of samples. The exact sample sizes of all datasets included in the training set are fully reported in the Materials and Methods section.

Following differential expression analysis of the training set, we generated heatmaps and volcano plots for core DEGs filtered by |log₂FC| > 1 and adjusted P-value < 0.05 (Fig 1). In the heatmap, blue and red denote low and high expression, respectively; the x-axis is ordered as “Control group → OA group (Treat)” with dataset labels, and the y-axis displays the top 50 upregulated and 50 downregulated genes. Results revealed a clear separation of expression profiles between groups: genes like *DDIT3*(log₂FC = −1.259) and *VEGFA* (log₂FC = −1.384) were highly expressed in controls but downregulated in OA, while genes such as *TREM1* (log₂FC = 1.675) and *COL1A1* (log₂FC = 1.671) showed the opposite trend. Consistent expression patterns of these DEGs across all five datasets suggested their potential involvement in OA pathological progression, providing promising targets for subsequent studies.

### GO and KEGG Enrichment Analyses of DEGs

**Fig 2:**
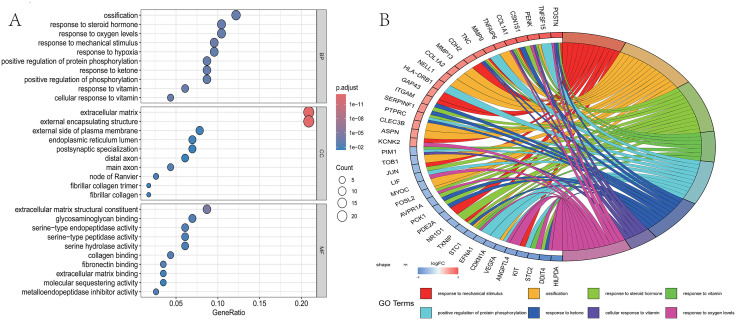
GO Enrichment Analysis of DEGs. (A) Bubble plot displaying the top significantly enriched GO terms categorized into BP, CC, and MF. Bubble size represents the number of DEGs per term; color gradient indicates the FDR-adjusted P-value (darker = higher significance). X-axis: gene ratio (DEG count in term/ total DEG count). (B) Chord diagram illustrating interactions between specific DEGs and enriched GO terms; chord width reflects the number of genes linking each pair. Enrichment was assessed using the hypergeometric test with Benjamini–Hochberg correction. Terms with adjusted P < 0.05 were considered significant. Abbreviations: DEG, differentially expressed gene; FDR, false discovery rate; GO, Gene Ontology.

DEGs underwent GO enrichment analysis, revealing strong associations with OA pathological features (Fig 2). In the BP category, DEGs were significantly enriched in ossification, response to mechanical stimulus, response to hormone/oxygen level stress, and regulation of protein phosphorylation. For CC, enrichment concentrated on extracellular matrix (ECM) and the external plasma membrane—structures aligning with OA’s hallmark cartilage matrix damage. The MF category highlighted ECM binding and serine-type endopeptidase activity, indicating DEGs act via matrix regulation or enzymatic activity.

Notably, key genes showed OA-specific enrichments: *MMP13*(pro-cartilage degradation) linked to “mechanical stimulus” and “ECM functions” [14]; *POSTN*(ossification-related) enriched in “ossification”; and *COL1A1*(collagen synthesis) involved in “ECM maintenance” [15]. Collectively, GO enrichment pinpoints core OA processes (stress response, ossification), cellular structures (ECM), and MF (matrix binding, enzyme activity) tightly tied to OA pathogenesis.

**Fig 3:**
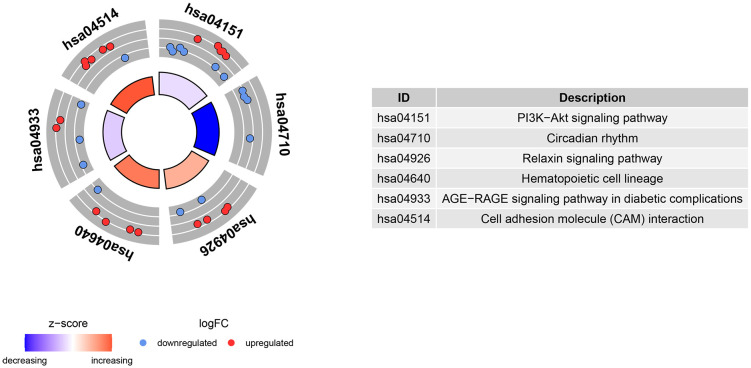
KEGG Pathway Enrichment Analysis. Circular plot displays the top six significantly enriched pathways. Outer sectors represent pathways labeled by KEGG ID; colored dots indicate individual genes (red = upregulated, blue = downregulated based on log2FC). Inner ring shows enrichment z-scores (blue = negative/down, orange = positive/up). Analysis was performed using the clusterProfiler R package via hypergeometric testing; pathways with adjusted P < 0.05 were deemed significant.

KEGG pathway enrichment analysis revealed that significantly DEGs were primarily enriched in 6 core pathways: PI3K-Akt signaling, circadian rhythm, relaxin signaling, hematopoietic cell lineage, AGE-RAGE signaling in diabetic complications, and cell adhesion molecules (CAMs) interaction. These pathways are closely linked to inflammatory responses, cell proliferation/apoptosis, matrix metabolism, and cell-cell interactions during OA pathological progression (Fig 3).

### Screening of Disease-Related Feature Genes

**Fig 4:**
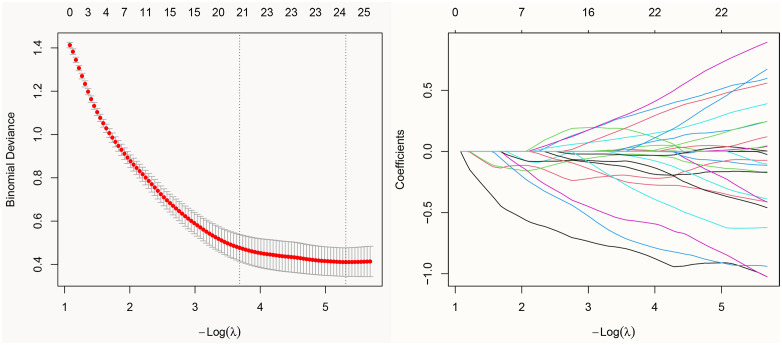
Least Absolute Shrinkage and Selection Operator (LASSO) Regression Screening. Model constructed using the glmnet R package. (A) Binomial deviance curve across log(λ) values. Optimal λ selected via 10-fold cross-validation at minimum error (dashed line). Top numbers indicate non-zero coefficient genes at each λ. (B) Coefficient profile plot showing gene coefficients vs. log(λ); each line = one gene. Genes retaining non-zero coefficients at optimal λ were retained as candidates.

**Fig 5:**
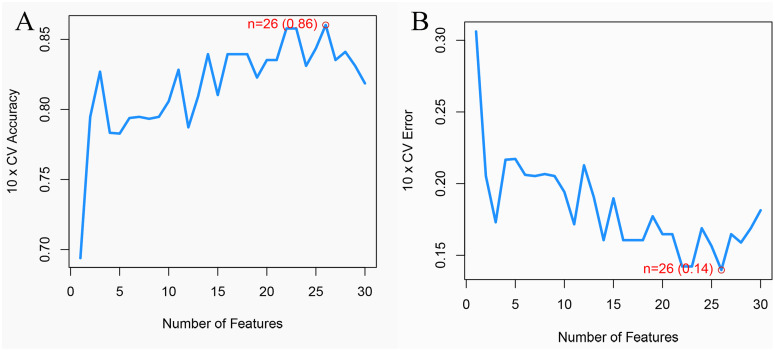
SVM-RFE Screening. Results of 10-fold cross-validation across varying feature counts. (A) Cross-validation accuracy curve; peak accuracy (0.86) achieved at 26 features. (B) Cross-validation error rate curve; minimum error (0.14) at 26 features. The optimal feature set was selected based on maximal accuracy and minimal error.

**Fig 6:**
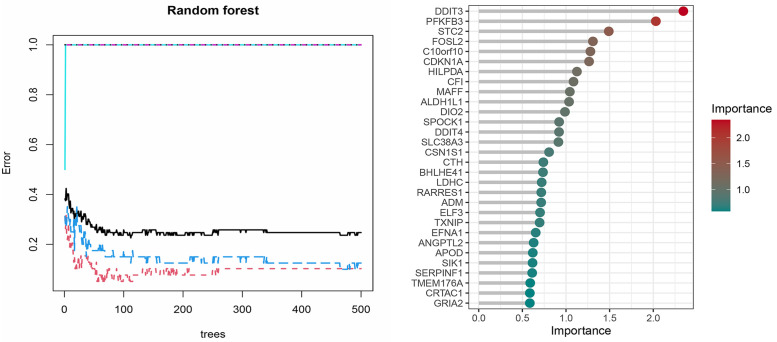
RF Algorithm Screening.

These significantly DEGs’ expression levels were analyzed using three machine learning algorithms—Lasso regression, RF, and SVM—to screen for candidate OA-related feature genes. For Lasso regression, as regularization strength decreased (-Log(λ) increased), the model’s binomial deviance first dropped then stabilized; the optimal λ (where fit and generalization balanced) retained core genes with non-zero coefficients, yielding 24 candidates (Fig 4). For SVM, plotting retained feature count (x-axis) against classification accuracy/error (y-axis) showed peak performance at 26 genes: 86% accuracy (Fig 5A) and 14% error (Fig 5B), selecting 26 core features. For RF, with ~300 decision trees, the error rate stabilized at a low level (Fig 6), indicating robust fit; gene importance analysis ranked *DDIT3, PFKFB3, STC2*, and *FOSL2* among top scorers, linking them to OA pathogenesis. These high-importance genes—24 from Lasso, 26 from SVM, and key RF-identified genes—serve as priority targets for subsequent OA molecular marker validation.

RF model trained with 500 trees using the randomForest R package. (Left) Out-of-bag (OOB) error rate vs. number of trees; curve stabilization indicates model convergence. (Right) Gene importance ranked by Mean Decrease Gini index. Genes with importance scores > 2 were selected as key candidates; *DDIT3* and *PFKFB3* showed the highest importance.

**Fig 7:**
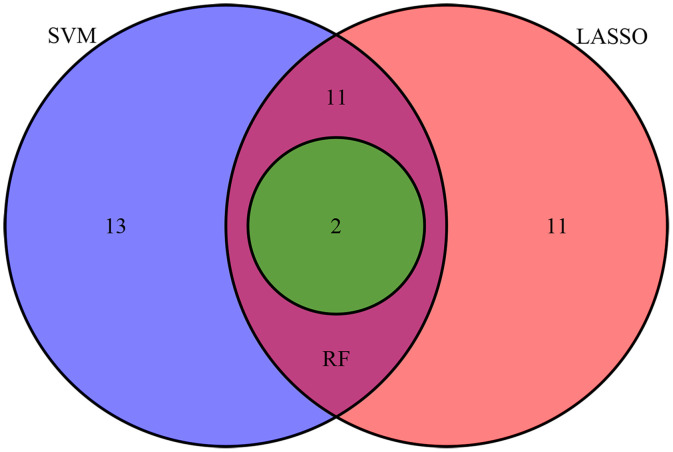
Intersection of Machine Learning Screening Results.

To obtain highly accurate and reliable disease-related feature genes, this study further integrated the screening results of three algorithms—LASSO regression, SVM-RFE, and Random Forest. Through intersection analysis of the gene sets selected by each algorithm, two core genes—*DDIT3* and *PFKFB3*—were ultimately identified (Fig 7).

Venn diagram displaying overlapping genes identified by LASSO regression, SVM-RFE, and Random Forest. The central intersection (2 genes) highlights the core feature genes selected by all three algorithms.

### Validation of Disease-Related Feature Genes in the Training Set

**Fig 8:**
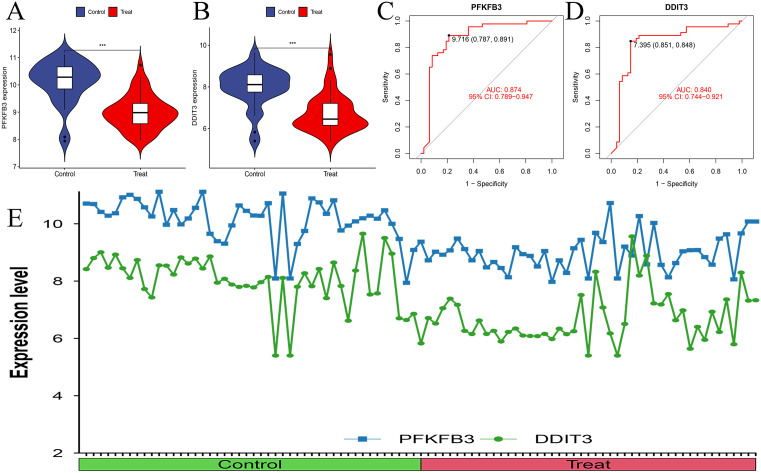
Expression Patterns and Diagnostic Performance of Core Genes in the Training Set. (A–B) Violin plots comparing *PFKFB3* and *DDIT3* expression between control and OA groups. Box inside violin represents median and interquartile range (IQR); whiskers extend to 1.5 × IQR. (C–D) ROC curves evaluating diagnostic accuracy; AUC with 95% CI shown. (E) Line plot of expression trends across samples. Statistical significance assessed using unpaired two-tailed Student’s t-test; P < 0.01.

To verify the expression differences and diagnostic value of the core feature genes *PFKFB3* and *DDIT3*, we performed violin plots, line plots, and ROC curve analyses. Violin plots (Fig 8A and 8B) revealed highly significant differences in *PFKFB3* and *DDIT3* expression between the control and OA groups (*** P < 0.001): both genes were highly expressed in controls and significantly downregulated in OA—consistent with prior differential analysis and RF results. Line plots (Fig 8E) visually displayed their expression trajectories: *PFKFB3* and *DDIT3* remained stably highly expressed in controls, while the OA group showed a consistent downward trend—further validating their group-specific patterns. ROC curve analysis (Fig 8C and 8D) showed *PFKFB3* had an AUC of 0.874 (95% CI: 0.789–0.947) with an optimal cutoff of 9.716 (sensitivity: 0.787; specificity: 0.891), and *DDIT3* had an AUC of 0.840 (95% CI: 0.744–0.921) with an optimal cutoff of 7.395 (sensitivity: 0.851; specificity: 0.848). Both genes exhibited AUC values greater than 0.8, indicating their excellent ability to distinguish between controls and OA patients and their potential as promising diagnostic molecular biomarkers for OA.

### Validation of Core Genes in the Validation Set

**Fig 9:**
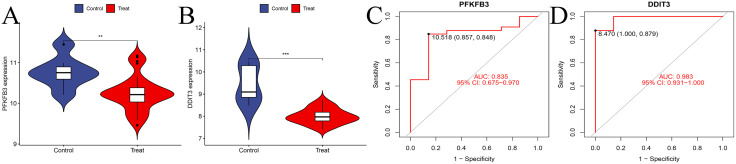
Validation of Core Genes in the Independent Cohort. (A–B) Violin plots of *PFKFB3* and *DDIT3* expression in control vs. OA groups (*P < 0.01, **P < 0.001). Data presentation and statistical test identical to Fig 8. (C–D) ROC curves with AUC and 95% CI for external validation.

To verify the expression differences and diagnostic value of the core feature genes *PFKFB3* and *DDIT3*, we performed violin plots, line plots, and ROC curve analyses. Violin plots (Fig 8A and 8B) revealed highly significant differences in *PFKFB3* and *DDIT3* expression between the control and OA groups (**P‌‌ < 0.001): both genes were highly expressed in controls and significantly downregulated in OA—consistent with prior differential analysis and RFresults. Line plots (Fig 8E) visually displayed their expression trajectories: *PFKFB3* and *DDIT3* remained stably highly expressed in controls, while the OA group showed a consistent downward trend—further validating their group-specific patterns. In the training set, ROC curve analysis (Fig 8C and 8D) showed *PFKFB3* had an AUC of 0.874 (95% CI: 0.789–0.947) with an optimal cutoff of 9.716 (sensitivity: 0.787; specificity: 0.891), and *DDIT3* had an AUC of 0.840 (95% CI: 0.744–0.921) with an optimal cutoff of 7.395 (sensitivity: 0.851; specificity: 0.848). Both genes exhibited AUC values greater than 0.8, indicating their robust discriminatory ability in the training cohort. To assess external generalizability, we further evaluated these metrics in an independent validation cohort (Fig 9A–9D). *DDIT3* yielded an AUC of 0.983 (95% CI: 0.931–1.000), while *PFKFB3* achieved an AUC of 0.835 (95% CI: 0.675–0.970). Notably, this validation set exhibited a marked class imbalance (33 OA vs. 7 controls). Although ROC-AUC is theoretically robust to prevalence shifts, the small total sample size combined with extreme imbalance may artificially inflate point estimates and widen CI, particularly for *DDIT3*. We therefore frame this validation result as preliminary evidence and caution against overinterpretation until confirmed in larger, demographically balanced cohorts.

### Results of Differential Expression and Co-Expression Analyses for Core Genes

**Fig 10:**
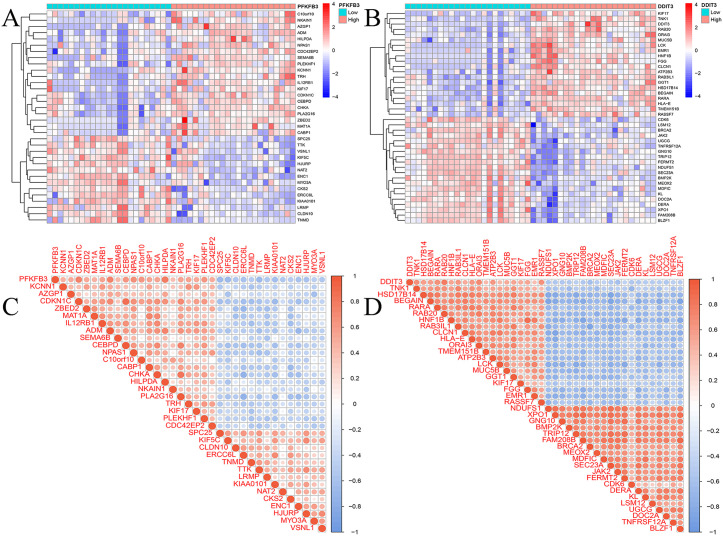
Co-Expression Analysis of Core Genes. (A–B) Heatmaps of transcripts correlated with *PFKFB3* and *DDIT3*. Rows represent genes and columns represent samples. The color gradient indicates normalized gene expression levels, with blue for low expression and red for high expression. (C–D) Bubble plots showing Spearman correlation coefficients between core genes and other transcripts. Bubble size corresponds to correlation strength, and bubble color represents statistical significance. All correlation analyses were performed using the Spearman correlation test, and a P-value less than 0.05 was defined as statistically significant.

To explore the functional roles of *DDIT3* and *PFKFB3* in OA, we stratified OA group samples into high- and low-expression subgroups based on the median expression levels of these two genes. Limma analysis (log₂FC > 1, adjusted P < 0.05) identified significantly DEGs between subgroups, and heatmaps visualized their expression patterns (Fig 10A–B)—showing clear clustering separation between high- and low-expression subgroups, which confirmed the reliability of the differential results. Further co-expression network analysis of *DDIT3* and *PFKFB3* (Fig 10C–D) revealed: *DDIT3* was strongly positively correlated with genes such as *HSD17B14*, *TNK1*, and *BEGAIN* (Pearson r ≥ 0.8), significantly negatively correlated with *UGCG*, *DOC2A*, *TNFRSF12A*, and *BLZF1* (r ≤ −0.8), and moderately negatively correlated with *CDK6* and *DERA* (r ≈ −0.6); *PFKFB3* exhibited strong positive correlations with *CHKA, CKS2,* and *ENC1* (r ≥ 0.8), significant negative correlations with *HJURP, MYO3A*, and *VSNL1* (r ≤ −0.8), and moderate positive correlations with *IL12RB1* and *CEBPD* (r ≈ 0.6). Together, these findings demonstrate that both core genes are associated with OA pathological progression through intricate positive and negative co-expression interactions with multiple functional genes.

### Immune Cell Infiltration and Immune Cell Correlation Analyses

**Fig 11:**
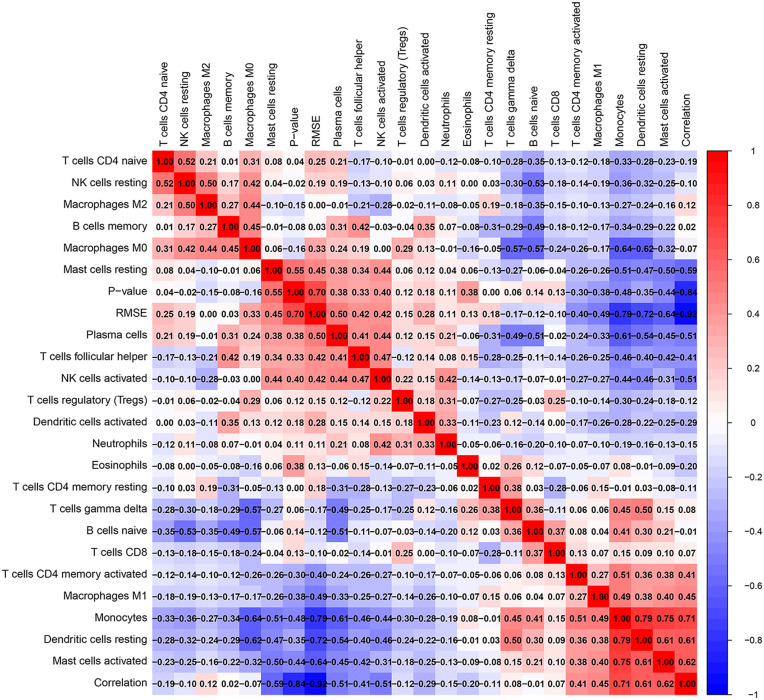
Immune Cell Infiltration Correlation Heatmap. Heatmap displaying pairwise correlation coefficients among 22 immune cell subtypes estimated by CIBERSORT. X-axis and Y-axis: 22 immune cell subtypes. Color intensity: correlation coefficient (r; red = positive, blue = negative). Numerical values or asterisks indicate statistical significance (P < 0.05, *P < 0.01, **P < 0.001) calculated using two-tailed Pearson correlation with FDR correction. Abbreviation: CIBERSORT, Cell-type Identification By Estimating Relative Subsets Of RNA Transcripts.

**Fig 12:**
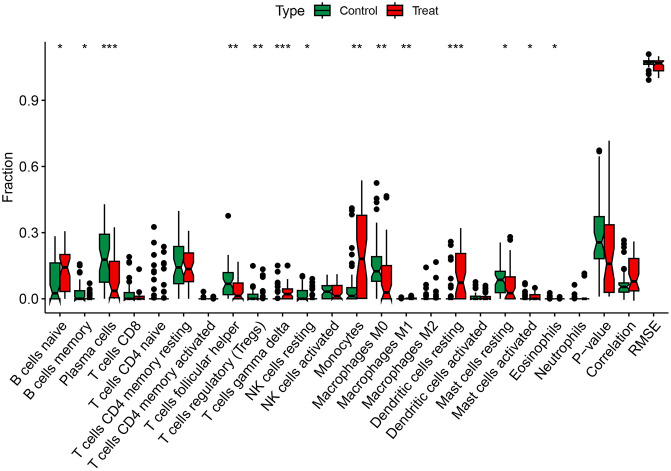
Differential Immune Cell Infiltration Between Groups. Box plots comparing relative proportions of 22 immune cell subtypes (estimated by CIBERSORT) between control and OA groups. Boxes denote median and IQR; whiskers extend to 1.5 × IQR; dots = individual samples. Statistical comparisons performed using the non-parametric Mann–Whitney U test. Significance levels: P < 0.05, *P < 0.01, **P < 0.001.

CIBERSORT-based deconvolution analysis revealed the presence of multiple immune cell subtypes in both control and OA samples, including B cells, T cell subsets, macrophages (M0/M1/M2), and natural killer (NK) cells. Notably, the algorithm-estimated relative proportions of these subsets differed significantly between groups (Fig 11). Box plot comparisons (Fig 12), analyzed using the non-parametric Mann–Whitney U test, confirmed significant intergroup differences in several populations, including macrophages and T cell subsets (P < 0.05, *P < 0.01, **P < 0.001). Within the OA cohort, correlation heatmap analysis revealed distinct co-infiltration patterns among immune cell subsets: resting NK cells showed a moderate positive correlation with M2 macrophages (r = 0.50); M0 macrophages were negatively correlated with memory B cells (r = −0.57); resting mast cells exhibited a strong negative correlation with eosinophils (r = −0.84); and monocytes were positively correlated with both resting dendritic cells and activated mast cells (r ≥ 0.75). These patterns suggest a coordinated reshaping of the local immune microenvironment in OA.

### Immune Relevance of Core Genes

**Fig 13:**
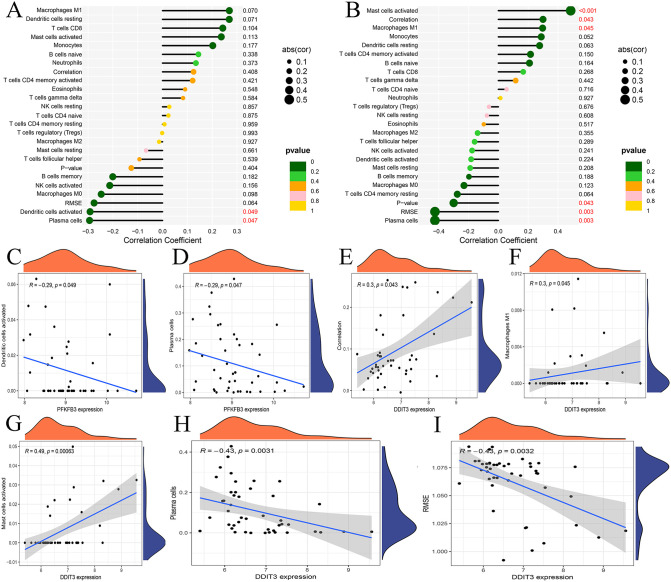
Association Between Core Genes and Immune Infiltration Profiles. (A–B) Lollipop plots showing correlation strength and significance between *PFKFB3*/*DDIT3* and immune cell subsets. (C–I) Scatter plots with marginal density distributions for specific gene–immune cell pairs: (C) *PFKFB3* vs. activated dendritic cells; (D) *PFKFB3* vs. plasma cells; (E) *DDIT3* vs. [Correlation indicator]; (F) *DDIT3* vs. M1 macrophages; (G) *DDIT3* vs. activated mast cells; (H) *DDIT3* vs. plasma cells; (I) *DDIT3* vs. root mean square error (RMSE). Correlation coefficients (r) and P-values were calculated using [Pearson/Spearman] tests. P < 0.05 considered significant.

To explore the association between *DDIT3/PFKFB3* expression and immune cell infiltration in OA group samples, we performed correlation analysis. Results showed *DDIT3* was significantly correlated with multiple immune cell types and analytical indicators (P < 0.05): it was strongly positively correlated with activated mast cells (r = 0.49, P = 0.00063), moderately positively correlated with M1 macrophages and a correlation-related indicator (both r = 0.30, P = 0.045 and 0.043, respectively), and strongly negatively correlated with plasma cells and the RMSE (Root Mean Square Error) indicator (both r = −0.43, P = 0.0031 and 0.0032, respectively). Additionally, *PFKFB3* exhibited statistically significant moderate negative correlations with activated dendritic cells (r = −0.29, P = 0.049) and plasma cells (r = −0.29, P = 0.047). Lollipop plots intuitively displayed the correlation strength and P-value distribution between *DDIT3/PFKFB3* and various immune cell types, while scatter plots with marginal densities further validated these significant linear trends (Fig 13).

### Results of ceRNA Network Analysis for Core Genes

**Fig 14:**
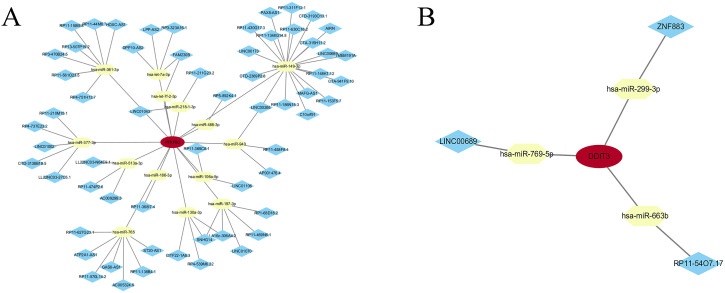
ceRNA Network Diagrams of Core GenesA: ceRNA network of PFKFB3; B: ceRNA network of DDIT3.

The results revealed that the core gene *DDIT3* targets three miRNAs—*hsa-miR-299-3p*, *hsa-miR-769-5p*, and *hsa-miR-663b*—while *lncRNA RP1–7.17* acts as a sponge for these miRNAs, thus potentially associated with *DDIT3* expression. For *PFKFB3*, we identified 16 high-confidence target miRNAs (e.g., *hsa-miR-130a-3p, hsa-let-7a-3p*) and 41 associated *lncRNAs (e.g., GAS6-AS1, SNHG14)*; these lncRNAs are potentially associated with *PFKFB3* expression via competitive miRNA sponging (Fig 14).

## Discussion

OA is a common chronic degenerative joint disease whose pathogenesis is intertwined with multiple factors such as age, obesity, joint injury, and genetics [16]. In-depth deciphering of its molecular mechanisms is crucial for developing effective preventive and therapeutic strategies. By integrating multiple machine learning algorithms and bioinformatics approaches, this study comprehensively mined multi-dimensional biological data and identified significant downregulation of *DDIT3* and *PFKFB3* in OA patients, providing novel insights into the molecular mechanisms and pathophysiological characteristics of OA.

This study clarified the specific co-expression patterns of *DDIT3* and *PFKFB3*, with the functions of their associated genes all pointing to core pathological processes of OA. *DDIT3* was strongly positively correlated with *HSD17B14, TNK1*, and *BEGAIN* (r ≥ 0.8) and significantly negatively correlated with *UGCG, DOC2A*, and *TNFRSF12A* (r ≤ −0.8): *HSD17B14* is associated with lipid metabolism, *TNK1* is associated with apoptotic signaling transduction [17], and *TNFRSF12A,* as a key molecule in inflammatory pathways [18], its abnormal expression may lead to joint tenderness, swelling, and mild elevations of inflammatory markers such as CRP and ESR in OA patients. These findings suggest that *DDIT3* may indirectly influence chondrocyte metabolism, apoptosis, and inflammatory responses through associations with these genes [19]. For *PFKFB3*, it was strongly positively correlated with *CHKA, CKS2*, and *ENC1*, and significantly negatively correlated with *HJURP* and *MYO3A. CHKA* is involved in phospholipid synthesis and cell survival, while *is associated with the cell cycle*; abnormalities in both functions are closely associated with chondrocyte dysfunction in OA [20,21]. Previous studies have confirmed that mRNA and protein expression of *PFKFB3* are significantly reduced in OA lesion tissues, accompanied by glycolytic metabolic abnormalities such as decreased glucose uptake, insufficient ATP production, and impaired lactate generation. Conversely, *PFKFB3* overexpression can improve chondrocyte survival via the PI3K-Akt/CHOP pathway, promote the synthesis of *Col II* and *ACAN*, inhibit the expression of *MMP13* and endoplasmic reticulum (ER) stress markers (*PERK, ATF3, IRE1, p-eIF2α*), and reduce *caspase 3* activation [20,21]. These results are consistent with the downregulation of *PFKFB3* in the OA group observed in this study, confirming that its low expression is associated with OA pathological progression by disrupting downstream co-expression networks. Notably, epigenetic analyses have shown that *PFKFB3* exhibits both downregulated expression and abnormal methylation, and together with *ADAMTS9* and *FKBP5*, it has been identified as a core methylation-related biomarker for OA, suggesting that its abnormal expression may be regulated by both genetic and epigenetic factors [22].

KEGG pathway enrichment analysis revealed that differentially expressed genes were concentrated in core OA-related pathways such as PI3K-Akt, AGE-RAGE, and Relaxin [23]. As a key associated with cell proliferation, apoptosis, and metabolism [24], the enrichment of the PI3K-Akt pathway suggests that *DDIT3* and *PFKFB3* may influence chondrocyte survival through this pathway. Previous studies have confirmed that *miR-130a* can activate the PI3K-Akt pathway by targeting *PTEN*, promoting chondrocyte proliferation and inhibiting apoptosis, while abnormal silencing of this pathway exacerbates OA progression [25], further supporting the molecular mechanism by which core genes are associated with OA pathology via the PI3K-Akt pathway. The AGE-RAGE pathway is associated with inflammatory responses induced by the accumulation of advanced glycation end products (AGEs). Elevated levels of AGEs have been detected in the synovial fluid of OA patients, which induce the release of inflammatory factors and accelerate cartilage matrix degradation by activating downstream signaling pathways [23]. Additionally, differentially expressed genes were enriched in the IL-17 signaling pathway, TNF signaling pathway, and osteoclast differentiation-related pathways, all of which have been confirmed to participate in OA pathogenesis by mediating inflammatory responses and regulating bone metabolic balance [26].

Immune infiltration analysis revealed significant differences in key immune cell populations such as macrophage subsets (M0/M1/M2), T cell subsets, and mast cells between the OA and control groups. The positive correlations between *DDIT3* expression and the estimated proportions of activated mast cells and M1 macrophages suggest a potential intersection between ER stress and innate immune activation in the OA microenvironment. *DDIT3* (also known as CHOP) is a terminal effector of the unfolded protein response (UPR) that drives apoptosis under prolonged cellular stress. Its co-variation with pro-inflammatory innate immune populations may reflect a stress-driven inflammatory feedback loop, wherein chondrocyte or synovial ER stress amplifies M1 macrophage polarization and mast cell degranulation, thereby exacerbating synovitis and cartilage matrix degradation. Conversely, the negative correlation with plasma cells could indicate a microenvironmental shift from adaptive/humoral immune regulation toward innate-driven inflammation as OA progresses, or alternatively, may reflect compartmentalized expression patterns where *DDIT3* downregulation coincides with niches enriched for antibody-producing cells. These associations align with emerging evidence linking UPR pathways to immune cell polarization and chronic joint inflammation, positioning *DDIT3* as a potential molecular node connecting cellular stress responses to immune remodeling in OA. Clinical studies have confirmed extensive immune cell infiltration in OA synovial tissues, accompanied by elevated levels of inflammatory factors such as *TNF-α* and *IL-1β* [25]. Correlation analysis between core genes and immune cells showed that *DDIT3* was strongly positively correlated with pro-inflammatory activated mast cells (r = 0.49, P = 0.00063), moderately positively correlated with M1 macrophages (r = 0.30, P = 0.045), and moderately negatively correlated with plasma cells (r = −0.43, P = 0.0031). *PFKFB3* exhibited moderately negative correlations with antigen-presenting activated dendritic cells (r = −0.29, P = 0.049) and plasma cells (r = −0.29, P = 0.047). M1 macrophages can induce low-grade local inflammation in joints via pathways such as *STAT, NF-κB*, and *MAPK*, disrupting chondrocyte metabolic balance and inhibiting cartilage repair processes [5,27]. Thus, the downregulation of *DDIT3* may reduce local inflammatory intensity and alleviate joint damage by weakening its positive regulation of M1 macrophages [5]. We speculate that the low expression of *DDIT3* is associated with immune status in the OA microenvironment. Together with *PFKFB3*, it shows correlations with the distribution of immune cell infiltration, which may help explain the individual differences in OA progression observed clinically. Studies on immune-related ceRNA networks have confirmed that differentially expressed lncRNAs in OA synovial tissues can participate in disease progression by regulating immune cell subset functions [20], further supporting the important role of the core gene-immune cell association axis in OA pathology.

ceRNA network analysis revealed the upstream association patterns underlying the abnormal expression of core genes. *DDIT3 is linked to* three “LncRNA-miRNA-*DDIT3*” pathways: *LINC00689* sponges *hsa-miR-769-5p*, *ZNF883* sponges *hsa-miR-299-3p*, and *RP11-54O7.17* sponges *hsa-miR-663b*, indirectly relieving the post-transcriptional inhibition of *DDIT3* by miRNAs. Similar networks are associated with chondrocyte functions in OA: for example, *LINC00689* affects chondrocyte apoptosis by competitively binding to miRNAs, and *hsa-miR-299-3p* is downregulated in OA cartilage, with abnormal activation of its target genes associated with matrix degradation [22]. We hypothesize that abnormal expression of lncRNAs impairs their sponging of miRNAs, enhancing the inhibitory effect of miRNAs on *DDIT3* and ultimately leading to its downregulation (which requires further experimental validation). The ceRNA network of *PFKFB3* is more complex, involving 41 lncRNAs such as *GAS6-AS1* and *SNHG14*, and 16 miRNAs such as *hsa-miR-130a-3p* and *hsa-let-7a-3p*. Among these, *GAS6-AS1* is associated with the expression of glycolysis-related genes in chondrocytes via the ceRNA mechanism [28], and *hsa-let-7a-3p* is highly expressed in OA cartilage, inhibiting target genes and causing cellular dysfunction [29,30], which is consistent with the clinical feature of abnormal glycolytic metabolism in OA chondrocytes. Genome-wide ceRNA network analyses have shown that lncRNA-miRNA-mRNA axes are widely involved in BP such as leukocyte migration and inflammatory signal activation in OA [31,32], further validating the pathological relevance of the core gene ceRNA networks.

Notably, the downregulation of *DDIT3* observed in this study is inconsistent with some reports suggesting that “high expression of *DDIT3* is positively correlated with OA cartilage damage” [33]. This discrepancy may be related to heterogeneity in sample characteristics and differential gene association networks of core genes, reflecting the complex and dynamic nature of OA pathogenesis. Downregulation of *DDIT3* is associated with reduced inflammatory activity and milder cartilage damage, which may correspond to clinical heterogeneity in symptom severity among OA patients. As the disease progresses, increased ER stress and inflammatory factor accumulation are associated with upregulation of *DDIT3*, which shows correlations with more severe cartilage degradation in temporomandibular joint OA. [33–35]. At this stage, patients experience persistent or nocturnal pain, with significant decline in joint function. The negative correlation between *DDIT3* and inflammatory genes such as *TNFRSF12A* further supports the potential link between its downregulation and reduced inflammatory activity in OA. In contrast, the downregulation of *PFKFB3* in this study is highly consistent with previous reports. Relevant studies have confirmed that low expression of *PFKFB3* in OA cartilage can cause glycolytic disorders and exacerbate ER stress, while its overexpression improves chondrocyte survival [20,36], which aligns with the results of this study showing that *PFKFB3* is associated with metabolism- and survival-related genes through co-expression networks, and is consistent with the clinical feature of abnormal energy metabolism in OA chondrocytes.

This study integrates five independent gene expression datasets with strict batch correction and three machine learning algorithms to reliably identify diagnostic genes for osteoarthritis. Compared with single‑dataset or single‑algorithm designs, this integrated approach improves the stability and reproducibility of feature gene selection. The systematic linkage of *DDIT3* and *PFKFB3* to ER stress, immune infiltration, glycolytic metabolism, and ceRNA regulation provides a more comprehensive landscape of OA pathogenesis, highlighting the unique value of these genes as potential diagnostic biomarkers and therapeutic targets. In conclusion, this study confirms that *DDIT3* and *PFKFB3* are significantly downregulated in OA patients. They are associated with metabolism- and inflammation-related co-expressed genes, participate in core pathways such as PI3K-Akt and AGE-RAGE, coordinately are associated with the balance of immune cell infiltration, and are linked to specific ceRNA networks and epigenetic modifications. The low expression of *PFKFB3* is associated with OA pathological progression via associations with glycolytic metabolic abnormalities and ER stress. Together, these two genes constitute key associated genes in OA pathology. This study provides potential biomarkers for the molecular diagnosis of OA and offers new theoretical bases and therapeutic targets for the development of targeted treatment strategies.

Although *DDIT3* and *PFKFB3* were identified as robust diagnostic candidates through integrated bioinformatic analyses, the present findings reflect statistical associations rather than established mechanistic regulation. Specifically, all immune cell infiltration profiles were computationally inferred using the CIBERSORT algorithm and should be interpreted as in silico‌‌ estimates rather than direct empirical measurements. Immune deconvolution of bulk cartilage transcriptomic data inherently carries limitations due to tissue heterogeneity, including the admixture of chondrocytes, stromal cells, and infiltrating immune populations. Consequently, these results are hypothesis-generating and require cautious interpretation, particularly given that disease stage–specific transcriptional shifts and spatial heterogeneity—unaccounted for in public datasets—may independently influence both gene expression and inferred immune landscapes. Similarly, the proposed link between *PFKFB3* and glycolytic reprogramming remains a computational inference; in the absence of direct functional metabolic assays, this association indicates potential involvement rather than experimentally validated regulatory control. Finally, the ceRNA axes involving *DDIT3* and *PFKFB3* are algorithmically predicted and biologically speculative until confirmed by orthogonal experimental approaches. Collectively, while these bioinformatic insights cannot substitute for mechanistic validation, they provide a prioritized, testable framework for future functional studies in chondrocyte homeostasis, ER stress, metabolic adaptation, and immune modulation in osteoarthritis.

However, this study has several limitations that should be acknowledged. First, the publicly available datasets used in this study lack complete clinical metadata, including age, sex, body mass index (BMI), and OA disease stage (e.g., Kellgren-Lawrence grade). These factors are well-known confounders in transcriptomic analyses and may significantly influence gene expression profiles. Since this information was unavailable in the source repositories, we could not adjust for these potential confounders, which may limit the generalizability of our findings. Importantly, any previous suggestion that DDIT3 downregulation might reflect early-stage OA was speculative and unsupported by the available metadata; it is therefore retracted. The observed differential expression should be interpreted as a cross-sectional association rather than a stage-specific biomarker. Future studies incorporating detailed demographic and phenotypic data are warranted to clarify the dynamic roles of *DDIT3* and *PFKFB3* throughout OA progression.
